# Predicting hospitalization following psychiatric crisis care using machine learning

**DOI:** 10.1186/s12911-020-01361-1

**Published:** 2020-12-10

**Authors:** Matthijs Blankers, Louk F. M. van der Post, Jack J. M. Dekker

**Affiliations:** 1 Department of Research, Arkin Mental Health Care, Klaprozenweg 111, 1033NN Amsterdam, The Netherlands; 2 Trimbos Institute, The Netherlands Institute of Mental Health and Addiction, Da Costakade 45, 3521VS Utrecht, The Netherlands; 3 Amsterdam UMC, Location AMC, Department of Psychiatry, University of Amsterdam, Meibergdreef 9, 1105AZ Amsterdam, The Netherlands; 4 Department of Clinical Psychology, VU University Amsterdam, Amsterdam, The Netherlands

**Keywords:** Psychiatric hospitalization, Machine learning, Acute psychiatry, Prognostic modeling

## Abstract

**Background:**

Accurate prediction models for whether patients on the verge of a psychiatric criseis need hospitalization are lacking and machine learning methods may help improve the accuracy of psychiatric hospitalization prediction models. In this paper we evaluate the accuracy of ten machine learning algorithms, including the generalized linear model (GLM/logistic regression) to predict psychiatric hospitalization in the first 12 months after a psychiatric crisis care contact. We also evaluate an ensemble model to optimize the accuracy and we explore individual predictors of hospitalization.

**Methods:**

Data from 2084 patients included in the longitudinal Amsterdam Study of Acute Psychiatry with at least one reported psychiatric crisis care contact were included. Target variable for the prediction models was whether the patient was hospitalized in the 12 months following inclusion. The predictive power of 39 variables related to patients’ socio-demographics, clinical characteristics and previous mental health care contacts was evaluated. The accuracy and area under the receiver operating characteristic curve (AUC) of the machine learning algorithms were compared and we also estimated the relative importance of each predictor variable. The best and least performing algorithms were compared with GLM/logistic regression using net reclassification improvement analysis and the five best performing algorithms were combined in an ensemble model using stacking.

**Results:**

All models performed above chance level. We found Gradient Boosting to be the best performing algorithm (AUC = 0.774) and K-Nearest Neighbors to be the least performing (AUC = 0.702). The performance of GLM/logistic regression (AUC = 0.76) was slightly above average among the tested algorithms. In a Net Reclassification Improvement analysis Gradient Boosting outperformed GLM/logistic regression by 2.9% and K-Nearest Neighbors by 11.3%. GLM/logistic regression outperformed K-Nearest Neighbors by 8.7%. Nine of the top-10 most important predictor variables were related to previous mental health care use.

**Conclusions:**

Gradient Boosting led to the highest predictive accuracy and AUC while GLM/logistic regression performed average among the tested algorithms. Although statistically significant, the magnitude of the differences between the machine learning algorithms was in most cases modest. The results show that a predictive accuracy similar to the best performing model can be achieved when combining multiple algorithms in an ensemble model.

## Background

In this paper, we evaluate and compare the performance of ten different machine learning (ML) algorithms to predict psychiatric hospitalization in the first 12 months after a psychiatric crisis care contact. Hospitalization is traditionally a preferred care modality for patients with severe mental illnesses or for those experiencing acute psychiatric crisis [1]. Recently, it has been debated whether hospitalization could be prescribed less often than has been done in the past, as in-patient acute mental health services are unpopular with service users [2, 3]. One of the reasons for this unpopularity is that hospitalization often fails to address individuals’ needs or to provide a safe and therapeutic environment [3-5]. Acute psychiatric hospitalization is also hypothesized to be more expensive than outpatient alternatives, although research on cost-effectiveness of alternatives to acute psychiatric hospitalization is still in its infancy [3]. Some patients however will still be hospitalized at some point during their illness and recovery. At this moment, it is difficult to predict which patient will be hospitalized in the near future, as currently a valid prognostic model for hospitalization after a psychiatric crisis is lacking.

### Related work

Previous studies (e.g. [6–9]) have done important work in identifying predictors (e.g. quality of life, psychiatric diagnosis, impact of symptoms, living situation) that can be relevant for a prognostic model for psychiatric hospitalization. Results show that previous (involuntary) admissions and the amount of previous psychiatric service use are reliable predictors of readmission [10–12]. Homelessness at admission discharge [11, 13], being on benefits [14], being unmarried, living alone or having a small social network [11, 15] and being of African and/or Caribbean origin [14] also are known predictors of psychiatric admission. Something these studies [6–15] have in common is that they used generalized linear modelling (GLM/logistic regression) for their prognostic models. Recently, papers have been published which have also used other modeling algorithms for their prognostic models, for example to predict re-hospitalization after heart failure [16], persistence of depression symptoms [17], or prediction of suicides after psychiatric hospitalisation [18]. Kessler and colleagues [17] found that other ML algorithms outperformed GLM/logistic regression in terms of model accuracy while in the two other studies [16, 18] the results of the different algorithms were quite similar. Hence, based on the evidence thus far we cannot conclude that specific ML algorithms consistently outperform others.

### Ensemble modeling and stacking

Ensemble modeling is a machine learning approach in which individual models are combined into one prediction model in order to improve the robustness and predictive accuracy of the final model [19]. Some ML algorithms, such as random forests, are based on the principles of ensemble modeling. However, with ensemble modeling it is also possible to combine different ML models. A common approach to create an ensemble model consisting of different ML algorithms is model stacking [20]. In model stacking, a meta-model uses the predicted outcomes of the prediction models as input instead of the variables in the dataset while the prediction target remains the same. The goal of stacking is to model under what circumstances each of the prediction models makes the most accurate prediction and to use this information in the prediction of the outcome variable [20].

### Aim and research questions

GLM and nine other ML algorithms were selected for the current prognostic modeling study in order to achieve maximum variation among the tested approaches. We will compare the performance of the ten algorithms in their ability to use a set of predictors to construct a prognostic model for psychiatric hospitalization following psychiatric crisis care, and we will evaluate whether an ensemble model of the best performing models created using stacking leads to more accurate predictions. We will use a routinely collected data set [19], containing similar variables as in some of the previously discussed studies. We will address the following questions:

1. Which of the evaluated ML algorithms have the best prognostic performance and does stacking further increase performance?
2. Which variables are the most powerful predictors for psychiatric hospitalization among patients on the verge of psychiatric crisis?
3. Which of the ML algorithms perform better than GLM/logistic regression in terms of predictive accuracy?

## Methods

### Patient data source

We will evaluate the ten ML algorithms using historical data from the Amsterdam Study of Acute Psychiatry (ASAP). The aim of ASAP was to study the association between the incidence of (involuntary) psychiatric hospitalizations and prior psychiatric history, the course of the psychiatric disorder, the patient’s social circumstances, and patient opinions and experiences [21, 22]. The dataset used in our study contains data from a cohort of patients who had an emergency consultation either by the Psychiatric Emergency Service Amsterdam or the Acute Treatment Unit in Amsterdam between 15 September 2004 and 15 September 2006 (the “index” contact). The patients were followed-up for 12 months. After the ASAP study, the intensive data collection was discontinued. Although some years old, this data set is still the largest, most extensive and complete dataset on long term hospitalization outcomes of psychiatric crisis care in the Netherlands.

### Predictor variables

The variables collected at baseline are age, gender, domestic situation and the Diagnostic and Statistical Manual of Mental Disorders, Fourth Edition, Text Revision axis I diagnosis. To determine the severity of psychopathology, the Severity of Psychiatric Illness rating scale (SPI) [23] was used. The SPI contains 14 items rated using a four-point scale: no risk, low risk, moderate risk, high risk—or no information present [24].

All variables related to health care consumption, and the number of care contacts in the 5 years before and the 12 months after the index contact were extracted from the patient health records kept by the three participating mental health institutions: JellinekMentrum (now Arkin), AMC de Meren (now Arkin), and GGZ inGeest. Table 1 presents the 39 predictor variables used to train our models.

**Table 1:** Predictor variables organized in three main themes

Sociodemographics	SPI items	Psychiatric care
Gender (cat)	Suicide risk (cat)	Patients’ informal social support system involved (cat)
Age (num)	Danger to others (cat)	Patient referrer (cat)
Living situation (cat)	Severity of psychiatric symptoms (cat)	Number of previous face-to-face treatment contacts up to 2 weeks/1 month/3 months/6 months/12 months before the index crisis care contact (num)
Marital status (cat)	Problems with self-care (cat)	Number of previous psychiatric hospitalizations (last 12 months and last 5 years) (num)
Cultural background (cat)	Substance misuse (cat)	Number of previous psychiatric day care treatments (last 12 months and last 5 years) (num)
Psychiatric diagnosis (cat)	Medical condition(s) (cat)	Number of involuntary treatments/hospitalizations (last 12 months and last 5 years) (num)
Global Assessment of Functioning (GAF) score (num)	Disturbances in patients’ family connectedness (cat)	Days of psychiatric hospitalization (last 12 months) (num)
	Professional functioning (cat)	Any earlier psychiatric care referrals (> 1 year and > 5 years before current contact) (num)
	Stability of patients’ living situation (cat)	
	Patient is motivated to receive treatment (cat)	
	Prescription medication compliance (cat)	
	Anosognosia (cat)	
	Patients’ family involvement in informal care (cat)	
	Symptom persistence (cat)	

*Data types cat* categorical data, *num* numerical data

All analyses were ran using routinely collected anonymized data from the participating institutions. Therefore, this study was exempted from medical ethics review and opt-in informed consent from participants was not necessary according to article 9 of the General Data Protection Regulation [25].

### Dependent variable

The dependent variable in our analysis was a dichotomized measure of hospitalization, operationalized as any psychiatric hospitalization in any of the three participating psychiatric hospitals in the 12 months after the index psychiatric crisis care contact.

### Machine learning algorithms

The ten ML algorithms evaluated in this paper are GLM/logistic regression, naive Bayes (R package klaR), stochastic gradient boosting (R package gbm), neural network (R package nnet), (model averaged) support vector machines with class weights (R package kernlab), k-nearest neighbors (R package class), (oblique) random forest (R packages randomForest and obliqueRF), DeepBoost (R package deepboost), and Keras/TensorFlow (R package keras and the TensorFlow and Keras libraries for Python). All algorithms had implementations in R and/or Python. The ML algorithms were chosen based on their dissimilarity in terms of modelling approaches and to represent the most commonly used types of algorithms for machine learning classification problems.

The *generalized linear model* (GLM) is a generalization of linear regression that allows for dependent variables to have error distributions other than a normal distribution. Using link functions, generalized linear models unify other statistical models such as linear regression, logistic regression and Poisson regression [26].

*Naive Bayes* is a technique for constructing models that classify cases into labels (in our case hospitalization Yes or No) based on a vector of case characteristics. Naive Bayes classifiers [27] assume that each characteristic is independent. For example, an animal can be considered a spider if it has eight legs, two body segments, and can produce silk. For a naive Bayes classifier each of these characteristics contributes independently to the probability that this animal is a spider, regardless of correlations between the characteristics.

*Gradient boosting* is an ML technique for regression and classification, which produces an ensemble of prediction models (often in the form of decision trees). It builds the model stage-wise and it generalizes them by optimizing a loss function [28, 29]. In Stochastic Gradient boosting, gradient boosting is combined with bootstrapping to improve the accuracy of the algorithm [26].

An (artificial) *neural network* is a ML model inspired by the biological neural networks such as in brains [30]. Neural networks are modelled to learn tasks based on provided examples, without being programmed with any task-specific rules. For example, neural networks might learn to identify images that contain spiders by analyzing example images that have been manually classified as “spider” or “no spider” and using the results to identify spiders in new images. In model averaged neural networks, the same neural network model is ran multiple times and the output from each run is averaged [31].

The *support vector machines* algorithm works by plotting each observation in n-dimensional space, where n is determined by the number of variables in the model [32]. The value of a case on each variable is the coordinate in the plotting space. Next, the algorithm performs classification by fitting a ‘hyperplane’ which optimally differentiates between the two classes. A hyperplane is an intersection of an n-dimensional space (with n − 1 dimensions). Using the parameters of the hyperplane, new observations can be classified.

*K-nearest neighbors* takes the k (k ≥ 1) closest matching examples from the training dataset in account, and assigns the majority class of these closest matching neighbors to the case that needs to be classified [33].

*Random forest* is an ensemble approach and in a sense similar to gradient boosting. Random forest algorithms produce many decision trees using the training data as an input. Each tree calculates values of the input variable which optimally split cases along the classes. A decision tree typically consists of multiple splits (nodes). Random forests use all those trees to predict class membership of a new case [34].

*Oblique random forests* distinguish themselves from ‘standard’ random forests by taking a multivariate approach to calculate each split [35], whereas the former uses one variable for each node.

*DeepBoost* is an ensemble learning algorithm, which optimizes the performance of other learning algorithms, which it uses to give optimally accurate classifications, while theoretically overcoming some of the limitations of other ensemble learning models [36].

As a final modelling approach, we used *TensorFlow*, an open source software library for numerical computation using data flow graphs. Nodes in the graph represent mathematical operations, while the graph edges represent the multidimensional data arrays (tensors) communicated between them. TensorFlow was originally developed by researchers and engineers working on the Google Brain Team within Google’s Machine Intelligence research organization for the purposes of conducting machine learning and deep neural networks research, but the system is general enough to be applicable in a wide variety of other domains as well [37]. We communicated with TensorFlow using the R package keras, which is an interface to Keras, the Python deep learning library capable of running on top of TensorFlow [38].

### Experimental procedures

First, a dataset was created consisting of the 39 predictor variables and the dependent variable. Data from patients with missing hospitalization data, missing SPI data, or from patients that died during the study’s follow-up period were removed. As some of the used statistical techniques cannot adequately handle missing data points, the remaining missing data were imputed using the mice package [39] with random forests in R.

All numeric predictor variables were centered and scaled in the pre-processing phase. Categorical variables were recoded into dummy variables. In the base case analysis, we have not applied balancing of the two levels of the dependent variable (hospitalized/not hospitalized); in a sensitivity analysis, all analyses were replicated under a balanced scenario which was created by under-sampling the most prevalent outcome. We used the default tuning hyperparameter optimisation approach in the R package caret [40], which is grid search. In this grid search, 3 different sets of values for the hyperparameters are evaluated; the best performing values are chosen for the final model. For each ML algorithm a corresponding grid search function is available in caret. An overview of the hyperparameters and the grid search functions per ML approach is included as Additional file 1. For TensorFlow, which is not available in caret, a custom grid search function was written, which has led to the optimisation of the number of dense layers and nodes. The final model has five dense layers with 4, 24, 8, 8, 68 nodes, and a 2 node output layer. We used the Adamax optimizer [41], the batch size of this model was 32 and the number of epochs was 16.

The ML algorithms were first applied to training data to parameterize and fit the model. Next, each model was validated using independent test data. We used K-fold cross-validation (with K = 10) to validate the model parameters. For K-fold cross-validation, K successive mutually exclusive test sets are created. Algorithm fitting is iteratively done on the training datasets. Predicted classifications are then calculated for the test set. With K = 10, at each iteration another 10% of the data is set aside from the original dataset for validation purposes. In the end, each observation in the original data set has a predicted classification that was obtained when it was part of the test set [42]. We chose K = 10 as a simulation study by Kohavi [43] indicated that for real word datasets the best method to use for model selection is tenfold stratified cross-validation.

Confusion matrices, accuracy scores, sensitivities, specificities and the Area under the Receiver Operating Characteristic (ROC) curves (AUC, or c-statistic) were calculated for each model. The AUC measures the area under the plot of the ROC curve and is an aggregate measure of the performance of the model [44]. Theoretically the AUC can have any value between 0 and 1, with 0 corresponding with 100% wrong predictions, and 1 corresponding with 100% correct predictions.

We also estimated the relative unique importance of each individual predictor variable for the overall AUC score using the filterVarImp function in the R package caret [40]. We standardized the AUC associated with each variable by dividing the absolute deviation for each variable by the absolute AUC deviation associated with the most impactful variable.

In order to evaluate the predictive accuracy of the most accurate model against the GLM-based model and against the least accurate model, we calculated the Net Reclassification Improvement (NRI). The NRI is an index that provides an estimate (with a confidence interval and a z-test) of how well a model classifies subjects compared to another model [45].

To evaluate the merits of ensemble modeling when predicting future psychiatric hospitalizations, we created a stacked (meta-)model comprising the five best performing ML models based on the calculated AUCs. For stacking, we have used the caret [40] package, in which we used the same preprocessing steps as we did for the underlying ML models. We used gradient boosting as the algorithm to create the stacked model and we used tenfold cross-validation to validate the meta-model parameters.

## Results

The original dataset contained data from 2707 patients. After removal of data from patients who had missing hospitalization data, completely missing SPI data, or who died during the follow-up period, data from 2084 patients remained. The completeness rate of this data set was high with only 4.2% missing data.

Table 2 presents some key characteristics of the full study sample (n = 2084) and for those hospitalized and not hospitalized in the year following the index contact separately. Based on chi-square tests, male participants have a higher probability of becoming hospitalized than female participants (37% vs. 31%, *p* = 0.001) and diagnosis (X^2^ = 120.1, *df* = 5; *p* < 0.0001), cultural background (X^2^ = 12.16, *df* = 5; p = 0.033) and living situation (X^2^ = 30.33; *df* = 5; *p* < 0.0001) are also associated with future hospitalization, while age (*p* = 0.33) is not (Table 2).

**Table 2:** Descriptive statistics for the 2084 patients in the first year after a psychiatric crisis care contact

Variable	All participants (n = 2084)	Hospitalized (n = 710)	Not hospitalized (n = 1374)	X^2^ (*df*)	*p*
	M (SD)|n (%)	M (SD)|n (%)	M (SD)|n (%)		
Age					
Years	40.8 (15.1)	41.0 (13.8)	40.7 (15.7)	0.94 (1)	0.33
Sex					
Male	1083 (52.0%)	405 (57.0%)	678 (49.3%)	10.81 (1)	0.001
Female	1001 (48.0%)	305 (43.0%)	696 (50.7%)		
Diagnosis				120.2 (5)	< 0.0001
Psychotic	807 (38.7%)	373 (52.5%)	434 (31.6%)		
Depressive	285 (13.7%)	98 (13.8%)	187 (13.6%)		
Substance related	239 (11.5%)	84 (11.8%)	155 (11.3%)		
Manic/bipolar	34 (1.6%)	12 (1.7%)	22 (1.6%)		
Other	561 (26.9%)	103 (14.5%)	458 (33.3%)		
No or deferred	158 (7.6%)	40 (5.6%)	118 (8.6%)		
Living situation				35.35 (5)	< 0.0001
Alone	1018 (48.8%)	385 (54.2%)	633 (46.1%)		
With partner/other(s)	564 (27.1%)	142 (20.0%)	422 (30.7%)		
With parents	235 (11.3%)	73 (10.3%)	162 (11.8%)		
Homeless	96 (4.6%)	42 (5.9%)	54 (3.9%)		
Institutionalized	68 (3.3%)	31 (4.4%)	37 (2.7%)		
Other	103 (4.9%)	37 (5.2%)	66 (4.8%)		
Cultural background				12.16 (5)	0.033
Dutch	1151 (55.2%)	409 (57.6%)	742 (54.0%)		
Surinamese/Antilles	303 (14.5%)	124 (17.5%)	189 (13.8%)		
Moroccan	145 (7.0%)	44 (6.2%)	101 (7.4%)		
Turkish	82 (4.0%)	22 (3.1%)	60 (4.4%)		
Other non-western	243 (11.7%)	78 (11.0%)	165 (12.0%)		
Other western	160 (7.7%)	43 (6.1%)	117 (8.5%)		

**Fig. 1:**
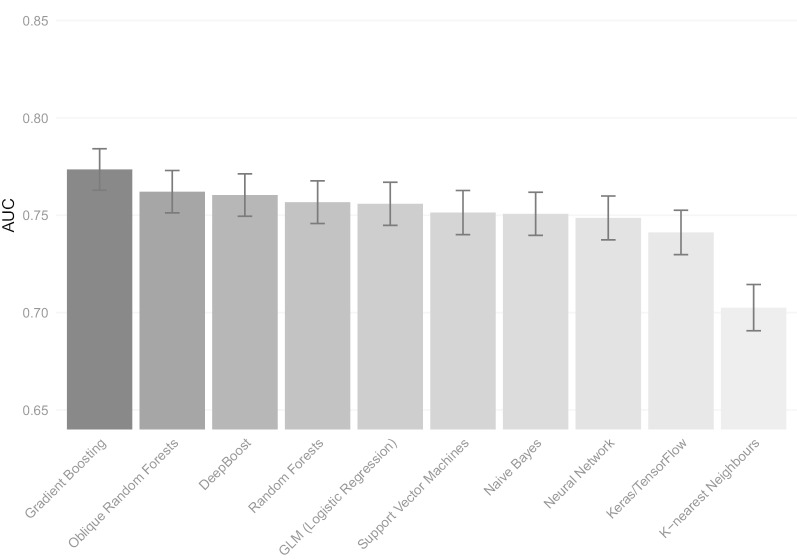
Comparison of AUC scores for the ten machine learning based models. *Note* AUC (or c-statistic) indicates the performance of the different machine learning based models. The error bars indicate ± 1 standard error intervals

Figure 1 presents the AUC statistics for the models using the ML algorithms based on the tenfold cross-validation tests using all 39 predictor variables. What can be observed foremost from Fig. 1 is that most confidence intervals of the models overlap. The Gradient Boosting-based model shows the best prognostic performance (AUC = 0.77), and K-Nearest Neighbors model has the least prognostic performance (AUC = 0.70). The performance of the GLM-based model is slightly above average (AUC = 0.76). The Gradient Boosting model also has the highest accuracy (0.744, see also Table 3). All models have an accuracy which is significantly above the ‘no information rate’ of 0.659, which is the proportion of not hospitalized patients in the dataset.

**Table 3:** Key performance statistics of the trained models

ML algorithm	AUC	Sensitivity	Specificity	Accuracy
Gradient boosting	0.774	0.455	0.894	0.744
Oblique random forest	0.762	0.509	0.847	0.732
DeepBoost	0.760	0.461	0.871	0.731
Random forest	0.757	0.478	0.864	0.732
GLM (logistic regression)	0.756	0.444	0.876	0.729
Support vector machines	0.751	0.370	0.917	0.731
Naive Bayes	0.751	0.455	0.861	0.723
Neural network	0.749	0.528	0.828	0.726
Keras/TensorFlow	0.741	0.465	0.850	0.719
K-nearest neighbors	0.702	0.356	0.879	0.701

The base rate of (non-)hospitalization = 0.659. The accuracy of each model was tested against this base rate, all *p* < 0.00001, based on 2-sided z-tests; hence each model led to a significant improvement in classification accuracy compared to an intercept only model

**Fig. 2:**
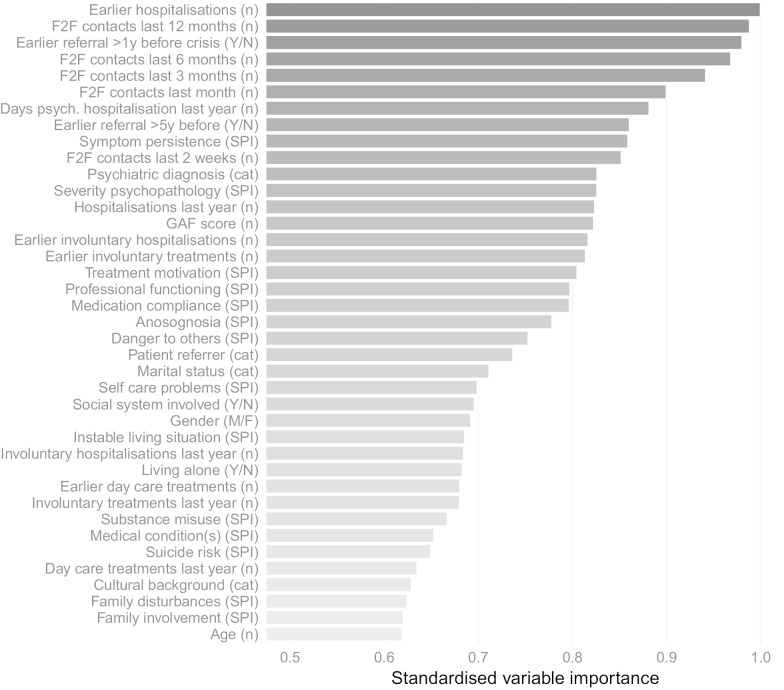
Overall variable importance plot for the machine learning based models. *Note* This plot presents the 39 predictors (before dummy-recoding) in descending order of unique predictive value. (n) indicates a numeric variable, (cat) indicates the variable is categorical, (SPI) indicates the variable is part of the SPI instrument, (M/F) and (Y/N) variables are dichotomous. Psychiatric care register data have a 5-year time horizon unless otherwise indicated

Figure 2 presents data on the relative importance of each variable for the AUC. Results are averaged over the ten models; in Additional File 2 we have presented the variable importance data for each model separately. Overall, it can be observed that the number of earlier psychiatric hospitalizations in the 5 years before the index contact and the number of face to face contacts the patient has had with professionals working for the participating mental health care center in the 12 months before have the strongest association with hospitalization in the year after the index contact.

In the NRI analysis the Gradient Boosting model led to 9.9% more correct classifications of hospitalized patients (z = 5.42, *p* < 0.0001) than the K-nearest Neighbors model and 1.5% more correct classifications of non-hospitalized patients, which was a non-significant improvement (z = 1.56, *p* = 0.12). Gradient Boosting led to an 11.3% increase in correctly classified patients overall in this comparison (z = 5.53, *p* < 0.0001).

Also the GLM/logistic regression model outperformed the K-nearest Neighbors model, and led to 8.7% more correct classifications of hospitalized patients (z = 4.64, *p* < 0.0001). The classification of not-hospitalized patients did not differ significantly between GLM/logistic regression and K-nearest Neighbors (− 0.3%, z = 0.31, *p* = 0.76). GLM/logistic regression led to an 8.4% increase in correctly classified patients overall in this comparison (z = 4.00, *p* < 0.0001).

Compared to GLM/Logistic regression, Gradient Boosting led to 1.1% more correct classifications of hospitalized patients (z = 0.88, *p* = 0.377), 1.8% more correct classifications of non-hospitalized patients (z = 2.57, *p* = 0.010), and a 2.9% increase in correctly classified patients overall (z = 1.99, *p* = 0.046).

In a final step, after training and evaluating models created using the individual ML algorithms, we have created a stacked model consisting of the models trained using Gradient Boosting, Oblique Random Forest, DeepBoost, Random Forest and GLM (Logistic Regression). The correlations between the outcomes of the five models were quite high (range 0.77–0.91). The final stacked model had an accuracy of 0.745, a sensitivity of 0.47 and a specificity of 0.89; the AUC was 0.764.

As a sensitivity analysis, we have performed all analyses on a balanced dataset as well, in which the prevalence of hospitalized and non-hospitalized patients was fixed to 0.5 and 0.5 respectively by under-sampling of the non-hospitalized patients. The results of the balanced dataset were very similar to those of the presented unbalanced dataset, including the differences between the most accurate algorithm (Gradient Boosting), GLM/Logistic regression and the least accurate algorithm (K-nearest Neighbors). Therefore, these results are not included in the main text but we included these as Additional File 3.

## Discussion

In this paper, we evaluated and compared the performance of prognostic models based on ten ML algorithms. We tested which models most accurately predicted hospitalization and which variables are the strongest predictors of psychiatric hospitalization.

All ten models had AUC scores > 0.7 and only three models (K-nearest Neigbors, TensorFlow, Neural Network) had an AUC < 0.75. There was no relevant difference between the models with regards to the AUC, except for the K-nearest neighbors algorithm which performed notably poorer than the other algorithms. Relative to the existing literature, these AUC scores could be considered high in the field of hospitalization prediction using clinical registry data. Artetxe and colleagues [46] in their overview of prediction models for hospital readmission in which they included 77 studies found that over 80% of the hospital readmission models in their review had an AUC score below 0.75—a finding in line with an earlier review by Kansagara et al. [47]. For clinical applications, an AUC of < 0.75 leaves room for improvement and often is of limited clinical utility.

We found differences in accuracy between the ML algorithms in this study, mostly of modest size: the absolute difference in accuracy between the best and least performing algorithm is only 0.04. We have compared the results of the best performing ML algorithm (Gradient Boosting) with logistic regression (GLM) and the least performing ML algorithm (K-Nearest Neighbors). We found that the Gradient Boosting model did outperform the GLM/logistic regression model and K-Nearest Neighbors model, and that the GLM/logistic regression model outperformed the K-Nearest Neighbors model in terms of classification accuracy. The reported differences in accuracy between the tested models are statistically significant but relatively small, especially for Gradient Boosting vs. GLM/logistic regression (2.9% difference in accuracy). This finding echoes the conclusion of the review by Artetxe and colleagues [46] that although promising, the real impact of recent ML algorithms in the domain of readmission risk prediction needs further study.

With regard to the importance of individual variables, we found that the number of earlier psychiatric hospitalizations in the last 5 years, the number of face to face therapy sessions in the last 12 months, and the number of earlier psychiatric care referrals > 12 months year before the initial crisis care contact were the strongest predictors of future psychiatric hospitalization. Nine out of the ten strongest predictors measured earlier mental health care consumption. Over the ten models there is some variation in which variables are the strongest predictors (see Additional File 2), although for all models the number of earlier hospitalizations is among the top three predictors, and the ten most impactful predictors are predominantly related to earlier mental health care.

In order to maximize the robustness and accuracy of our final prediction model, we have created a stacked model comprising the five best performing individual ML models. The accuracy and AUC of the stacked model was almost identical to the Gradient Boosting model and we therefore conclude that in this study no improvement in accuracy was achieved by stacking multiple prediction models. This may be related to the high correlations between the outcomes of the individual prediction models (0.77 and higher).

### Strengths and limitations

The findings of this study should be interpreted in the light of its strengths and limitations. A strength of this study is the relatively large clinical dataset of 2084 patients from which 710 patients were hospitalized during the follow-up period, and the availability of 39 clinically relevant potential predictors of hospitalization. For psychiatry crisis care research projects it is rare to achieve such sample sizes. Another strength is that the dataset consists of routinely collected clinical ‘real life’ data with high ecological validity, while missing data rates are modest (4.2%). Methodological strengths of this study include the direct comparison of ten different ML algorithms, and the use of K-fold cross-validation to optimally use the available data to train and test the models [42, 43].

Limitations of our study are the fact that although the average missing data rate after data selection was quite low we still had to address data missingness via imputation, as most ML algorithms are not capable of working with data with missing observations. Another limitation is that although we have made a diverse selection of ML algorithms, it is a matter of debate to what extent findings regarding the selected algorithms generalize to other algorithms; there is a possibility that better results could have been achieved with other ML algorithms. A third limitation is that the number of variables available in the dataset was—for ML purposes—somewhat limited. A forth limitation is that regarding the impact of the individual variables, only the unique variance explained by each variable could be assessed. This may have led to an underestimation of the importance of some variables when algorithms which are less well able to handle correlated predictors such as GLM were applied. A further limitation is that not all algorithms were similarly well equipped to discover relevant interactions in a data driven manner between predictor variables—this may be part of an explanation for differences in accuracy between the algorithms. Lastly, we do not know to what extend our findings related to the (non-)superiority of some algorithms over others generalize well beyond the context in which we evaluated them.

### Implications

One of the findings of this study is that there may be slightly more accurate algorithms than GLM/logistic regression to develop a prognostic model for future psychiatric hospitalization—although the potential gains in accuracy are limited in clinical impact. We found Gradient Boosting to outperform the other individual algorithms in this analysis, and an ensemble model based on the 5 best performing algorithms to perform similarly, but we do not know whether this finding generalizes beyond our study.

As long as a more definitive and validated answer is lacking to what the most accurate algorithm is and under what conditions, one could use multiple ML algorithms in an ensemble when creating a prognostic model. In this way, the risk of relying on a poorly performing algorithm is mitigated. More research is needed to evaluate which set of ML algorithms performs optimally when combined in an ensemble model.

Regarding the clinical implications, we can conclude that although the differences between the models was small, we were able to create an ensemble model with an overall 74.5% accuracy and 89% specificity to predict future psychiatric hospitalizations on unseen data (i.e. the test dataset). Although the sensitivity was quite low (47%), the specificity score means that from every 100 patients for which our model indicates that he or she will not be hospitalized, 89 patient will in fact not become hospitalized in the next year. Potentially, this classification model therefore has clinical utility. We do not yet know to what extent the algorithms perform equally well among subgroups of patients, e.g. old versus young, men versus women, or among patient groups with different diagnoses. Future research should also evaluate how well this model performs compared to human raters, and whether it is feasible to integrate an automated prediction model in the clinical practice of acute psychiatry.

## Conclusions

In this paper, we showed it is feasible to construct a prognostic model for psychiatric hospitalization with an acceptable AUC, accuracy and specificity compared to previous studies, using the predictors we evaluated. Variables on previous mental health care consumption were the strongest predictors of psychiatric hospitalization. Gradient Boosting led to the highest predictive accuracy and AUC, and GLM/logistic regression performed average compared to the other algorithms. An ensemble model comprising the five best models performed similar to the Gradient Boosting model. Although statistically significant, we conclude that the improvement of the best performing algorithm over GLM/logistic regression is limited. We also found that the difference in predictive performance between the best and least performing model is modest. Future studies may shed light on how ensemble models could be of practical value in the field of acute psychiatry.

## Supplementary Information

**Additional file 1.** Default grid search settings caret package.

**Additional file 2.** Variable importance data separately for each model.

**Additional file 3.** Key results of our sensitivity analysis using balanced data.

## Data Availability

The data that support the findings of this study are available from Arkin Mental Health Care and GGZ inGeest but restrictions apply to the availability of these data, which were used under license for the current study, and so are not publicly available. Data are however available from the authors upon reasonable request and with permission of Arkin Mental Health Care and GGZ inGeest.

## Abbreviations

ASAP: Amsterdam Study of Acute Psychiatry
AUC: Area under the receiver operating characteristic curve
GAF: Global Assessment of Functioning
GLM: Generalized linear modelling
ML: Machine learning
NRI: Net reclassification improvement
ROC: Receiver operating characteristic
SPI: Severity of Psychiatric Illness rating scale
