# Supplementary material for: Predicting hospitalization following psychiatric crisis care using machine learning
Source: BMC Med Inform Decis Mak. 2020 Dec 10;20:332. doi: 10.1186/s12911-020-01361-1 (PMC7731561; doi:10.1186/s12911-020-01361-1)
Supplement: Supplementary file 1 — Additional file 1. Default grid search settings caret package. [file 12911_2020_1361_MOESM1_ESM.pdf]

```r
### Default grid search settings caret package
### Matthijs Blankers
### 27 August 2019

library(caret)
```

```
## Loading required package: lattice

## Loading required package: ggplot2
```

```r
packageVersion("caret")
```

```
## [1] '6.0.80'
```

```r
get <- c("parameters", "grid")

getModelInfo("glm")$glm[get]
```

```
## $parameters
##   parameter     class     label
## 1 parameter character parameter
##
## $grid
## function (x, y, len = NULL, search = "grid")
## data.frame(parameter = "none")
```

```r
getModelInfo("nb")$nb[get]
```

```
## $parameters
##   parameter   class                label
## 1        fL numeric   Laplace Correction
## 2 usekernel logical    Distribution Type
## 3    adjust numeric Bandwidth Adjustment
##
## $grid
## function (x, y, len = NULL, search = "grid")
## expand.grid(usekernel = c(TRUE, FALSE), fL = 0, adjust = 1)
```

```r
getModelInfo("gbm")$gbm[get]
```

```
## $parameters
##           parameter   class                   label
## 1           n.trees numeric   # Boosting Iterations
## 2 interaction.depth numeric          Max Tree Depth
## 3         shrinkage numeric               Shrinkage
## 4    n.minobsinnode numeric Min. Terminal Node Size
##
## $grid
## function (x, y, len = NULL, search = "grid")
## {
##     if (search == "grid") {
```

```
##         out <- expand.grid(interaction.depth = seq(1, len), n.trees = floor((1:len) *
##             50), shrinkage = 0.1, n.minobsinnode = 10)
##     }
##     else {
##         out <- data.frame(n.trees = floor(runif(len, min = 1,
##             max = 5000)), interaction.depth = sample(1:10, replace = TRUE,
##             size = len), shrinkage = runif(len, min = 0.001,
##             max = 0.6), n.minobsinnode = sample(5:25, replace = TRUE,
##             size = len))
##         out <- out[!duplicated(out), ]
##     }
##     out
## }
```

```
getModelInfo("avNNet")$avNNet[get]
```

```
## $parameters
##   parameter   class         label
## 1      size numeric #Hidden Units
## 2     decay numeric  Weight Decay
## 3       bag logical       Bagging
##
## $grid
## function (x, y, len = NULL, search = "grid")
## {
##     if (search == "grid") {
##         out <- expand.grid(size = ((1:len) * 2) - 1, decay = c(0,
##             10^seq(-1, -4, length = len - 1)), bag = FALSE)
##     }
##     else {
##         out <- data.frame(size = sample(1:20, size = len, replace = TRUE),
##             decay = 10^runif(len, min = -5, 1), bag = sample(c(TRUE,
##                 FALSE), size = len, replace = TRUE))
##     }
##     out
## }
```

```
getModelInfo("svmRadialWeights")$svmRadialWeights[get]
```

```
## $parameters
##   parameter   class  label
## 1     sigma numeric  Sigma
## 2         C numeric   Cost
## 3    Weight numeric Weight
##
## $grid
## function (x, y, len = NULL, search = "grid")
## {
##     sigmas <- kernlab::sigest(as.matrix(x), na.action = na.omit,
##         scaled = TRUE)
##     if (search == "grid") {
##         out <- expand.grid(sigma = mean(as.vector(sigmas[-2])),
##             C = 2^((1:len) - 3), Weight = 1:len)
##     }
##     else {
```

```
##         rng <- extendrange(log(sigmas), f = 0.75)
##         out <- data.frame(sigma = exp(runif(len, min = rng[1],
##             max = rng[2])), C = 2^runif(len, min = -5, max = 10),
##             Weight = runif(len, min = 1, max = 25))
##     }
##     out
## }
```

```r
getModelInfo("knn")$knn[get]
```

```
## $parameters
##   parameter   class      label
## 1         k numeric #Neighbors
##
## $grid
## function (x, y, len = NULL, search = "grid")
## {
##     if (search == "grid") {
##         out <- data.frame(k = (5:((2 * len) + 4))[(5:((2 * len) +
##             4))%%2 > 0])
##     }
##     else {
##         by_val <- if (is.factor(y))
##             length(levels(y))
##         else 1
##         out <- data.frame(k = sample(seq(1, floor(nrow(x)/3),
##             by = by_val), size = len, replace = TRUE))
##     }
##     out
## }
```

```r
getModelInfo("rf")$rf[get]
```

```
## $parameters
##   parameter   class                         label
## 1      mtry numeric #Randomly Selected Predictors
##
## $grid
## function (x, y, len = NULL, search = "grid")
## {
##     if (search == "grid") {
##         out <- data.frame(mtry = caret::var_seq(p = ncol(x),
##             classification = is.factor(y), len = len))
##     }
##     else {
##         out <- data.frame(mtry = unique(sample(1:ncol(x), size = len,
##             replace = TRUE)))
##     }
##     out
## }
```

```r
getModelInfo("deepboost")$deepboost[get]
```

```
## $parameters
##    parameter     class                     label
## 1   num_iter   numeric     # Boosting Iterations
```

```
## 2 tree_depth   numeric                Tree Depth
## 3       beta   numeric         L1 Regularization
## 4     lambda   numeric Tree Depth Regularization
## 5  loss_type character                      Loss
##
## $grid
## function (x, y, len = NULL, search = "grid")
## {
##     if (search == "grid") {
##         out <- expand.grid(tree_depth = seq(1, len), num_iter = floor((1:len) *
##             50), beta = 2^seq(-8, -4, length = len), lambda = 2^seq(-6,
##             -2, length = len), loss_type = "l")
##     }
##     else {
##         out <- data.frame(num_iter = floor(runif(len, min = 1,
##             max = 500)), tree_depth = sample(1:20, replace = TRUE,
##             size = len), beta = runif(len, max = 0.25), lambda = runif(len,
##             max = 0.25), loss_type = sample(c("l"), replace = TRUE,
##             size = len))
##     }
##     out
## }
```

```r
getModelInfo("ORFpls")$ORFpls[get]
```

```
## $parameters
##   parameter   class                         label
## 1      mtry numeric #Randomly Selected Predictors
##
## $grid
## function (x, y, len = NULL, search = "grid")
## {
##     if (search == "grid") {
##         out <- data.frame(mtry = caret::var_seq(p = ncol(x),
##             classification = is.factor(y), len = len))
##     }
##     else {
##         out <- data.frame(mtry = unique(sample(1:ncol(x), size = len,
##             replace = TRUE)))
##     }
##     out
## }
```
