# Supplementary material for: Predicting hospitalization following psychiatric crisis care using machine learning
Source: BMC Med Inform Decis Mak. 2020 Dec 10;20:332. doi: 10.1186/s12911-020-01361-1 (PMC7731561; doi:10.1186/s12911-020-01361-1)
Supplement: Supplementary file 3 — Additional file 3. Key results of our sensitivity analysis using balanced data. [file 12911_2020_1361_MOESM3_ESM.docx]

**Additional File 3: Key results of sensitivity analysis using balanced data**

**Matthijs Blankers, Louk F. M. van der Post, Jack J. M. Dekker**

*Figure 1. Comparison of AUC scores for the ten machine learning based models*

**

*Note. AUC (or c-statistic) indicates the performance of the ten models based on the machine learning algorithms. The error bars indicate +/- 1 standard error intervals.*

*Figure 2: Overall variable importance plot for the machine learning based models*

**

*Note. This plot presents the 39 predictors (before dummy-recoding) in descending order of unique predictive value. (n) indicates a numeric variable, (cat) indicates the variable is categorical, (SPI) indicates the variable is part of the SPI instrument, (M/F) and (Y/N) variables are dichotomous. Psychiatric care register data have a 5-year time horizon unless otherwise indicated.*

*Net Reclassification Improvement Gradient Boosting vs. K-nearest Neighbors*

## Proportions of Subjects with Improvement in Predicted Probability

##

## Number of events: 710 Number of non-events: 710

##

## Proportions of Positive and Negative Changes in Probabilities

##

## Proportion

## Increase for events (1) 0.1563

## Increase for non-events (2) 0.0986

## Decrease for events (3) 0.0915

## Decrease for non-events (4) 0.1521

##

##

## Net Reclassification Improvement

##

## Index SE Z 2P Lower 0.95 Upper 0.95

## NRI (1-3+4-2) 0.1183 0.0263 4.50 6.91e-06 0.0667 0.1699

## NRI events (1-3) 0.0648 0.0185 3.50 4.70e-04 0.0285 0.1011

## NRI non-events (4-2) 0.0535 0.0187 2.86 4.17e-03 0.0169 0.0901

##

*Net Reclassification Improvement GLM/logistic regression vs. K-nearest Neighbors*

## Proportions of Subjects with Improvement in Predicted Probability

##

## Number of events: 710 Number of non-events: 710

##

## Proportions of Positive and Negative Changes in Probabilities

##

## Proportion

## Increase for events (1) 0.1310

## Increase for non-events (2) 0.1028

## Decrease for events (3) 0.0958

## Decrease for non-events (4) 0.1338

##

##

## Net Reclassification Improvement

##

## Index SE Z 2P Lower 0.95 Upper 0.95

## NRI (1-3+4-2) 0.0662 0.0255 2.60 0.00939 0.01625 0.1161

## NRI events (1-3) 0.0352 0.0178 1.98 0.04819 0.00028 0.0701

## NRI non-events (4-2) 0.0310 0.0182 1.70 0.08898 -0.00472 0.0667

##

*Net Reclassification Improvement Gradient Boosting vs. GLM/logistic regression*

## Proportions of Subjects with Improvement in Predicted Probability

##

## Number of events: 710 Number of non-events: 710

##

## Proportions of Positive and Negative Changes in Probabilities

##

## Proportion

## Increase for events (1) 0.0704

## Increase for non-events (2) 0.0577

## Decrease for events (3) 0.0408

## Decrease for non-events (4) 0.0803

##

##

## Net Reclassification Improvement

##

## Index SE Z 2P Lower 0.95 Upper 0.95

## NRI (1-3+4-2) 0.0521 0.0187 2.79 0.00529 0.01549 0.0887

## NRI events (1-3) 0.0296 0.0125 2.37 0.01769 0.00514 0.0540

## NRI non-events (4-2) 0.0225 0.0139 1.62 0.10540 -0.00474 0.0498

##
